# Analysis of Plasma Using Flow Cytometry Reveals Increased Immune Cell-Derived Extracellular Vesicles in Untreated Relapsing-Remitting Multiple Sclerosis

**DOI:** 10.3389/fimmu.2022.803921

**Published:** 2022-03-22

**Authors:** Stephanie N. Blandford, Neva J. Fudge, Chris P. Corkum, Craig S. Moore

**Affiliations:** Division of Biomedical Sciences, Faculty of Medicine, Memorial University of Newfoundland, St. John’s, NL, Canada

**Keywords:** extracellular vesicles, biomarkers, immunology, multiple sclerosis, plasma

## Abstract

Extracellular vesicles (EVs) are secreted from cells under physiological and pathological conditions, and are found in biological fluids while displaying specific surface markers that are indicative of their cell of origin. EVs have emerged as important signaling entities that may serve as putative biomarkers for various neurological conditions, including multiple sclerosis (MS). The objective of this study was to measure and compare immune cell-derived EVs within human plasma between untreated RRMS patients and healthy controls. Using blood plasma and peripheral blood mononuclear cells (PBMCs) collected from RRMS patients and controls, PBMCs and EVs were stained and quantified by flow cytometry using antibodies against CD9, CD61, CD45, CD3, CD4, CD8, CD14, and CD19. While several immune cell-derived EVs, including CD3^+^, CD4^+^, CD8^+^, CD14^+^, and CD19^+^ were significantly increased in RRMS vs. controls, no differences in immune cell subsets were observed with the exception of increased circulating CD19^+^ cells in RRMS patients. Our study demonstrated that plasma-derived EVs secreted from T cells, B cells, and monocytes were elevated in untreated RRMS cases with low disability, despite very limited changes in circulating immune cells, and suggest the utility of circulating EVs as biomarkers in MS.

## Introduction

Extracellular vesicles (EVs) are small (~30-200nm) biological vesicles continuously secreted from cells under both physiological and pathological conditions (1, 2). Secreted EVs can be detected within all biological fluids and have numerous functions, however, they have only recently emerged as an important mechanism of intercellular communication (1, 3). EVs contain biologically active cargo, which can be either non-specific or unique to the cell of origin (4, 5). The presence of cell-specific markers on the surface of EVs allows for the identification of the EVs’ cell type of origin and permits their quantification using specialized instrumentation and standardized assays (6). EVs of various cellular origins have emerged as important signaling entities that may help to drive pathophysiological conditions and could serve as putative biomarkers for various neurological conditions (1, 2).

Multiple sclerosis (MS) is a chronic inflammatory neurological disease characterized by the immune-mediated destruction of myelin that results in impaired neurotransmission and subsequent neurodegeneration (7). The search for easily accessible, non-invasive, and cost-effective biomarkers for complex CNS conditions, including MS, have proved difficult to clinically validate. In MS, current clinically useful biomarkers require expensive equipment (e.g. MRI, Simoa™) and/or CSF analyses obtained from an invasive lumbar puncture (8). Despite decades of investigation, an easily detectable blood-based biomarker for MS has yet to be fully adopted by the field; blood-derived EVs have the potential to bridge this gap. Unique immune cell-derived EV signatures (and their associated cargo) may provide valuable insights into the phenotype and activity/function of immune cells and help to further elucidate how they are contributing to ongoing pathological processes.

In MS, early studies focused on the secretion of EVs from endothelial cells and platelets due to their relatively high abundance within human blood. These studies demonstrated significantly increased levels of total EV particles (including exosomes, microparticles, and apoptotic bodies), particularly in clinically active cases (9–15). In more recent years, a focus has shifted to investigate particles secreted from major effectors of MS pathology, including T cells, B cells, and monocytes (14, 16, 17). When investigating these specific populations, the published literature has been inconsistent due to small sample sizes, and differences in staining and flow cytometry parameters, assays, and instruments.

The objectives of this study were to provide a comprehensive evaluation of circulating EVs secreted from cells that are of interest to MS pathophysiology, namely T cells, monocytes, and B cells, and compare them to healthy controls. We also aimed to determine whether any changes observed in EV populations were related to any overall changes in circulating lymphocyte populations within an individual.

## Materials and Methods

### Human Sample Collection and Preparation

Protocols and experiments involving human participants were approved by the Newfoundland Health Research Ethics Board. Written informed consent was obtained from all participants prior to study initiation. MS patients were diagnosed according to 2017 McDonald criteria (18) and recruited through the Health Research Innovation Team in Multiple Sclerosis (HITMS), an MS patient registry and biorepository at Memorial University of Newfoundland, St. John’s NL, Canada between February 2016 and January 2021. Patient plasma samples were chosen based on sample availability; the only exclusion criteria considered was use of disease-modifying therapies (DMT) (including steroids) within 12 months of sample collection. This study consisted of 33 DMT free relapse remitting MS (RRMS) patients and 22 age- and sex-matched healthy controls (see **Table 1**). All healthy control samples used in this study were obtained from healthy donors with no prior history of systemic diseases or administration of any immunosuppressing or immunomodulatory drugs. In the MS cohort, 20 patients had no previous history of DMT use, 6 had previously been on interferon beta-1a and discontinued 6-12 years prior to sample collection, 3 had previously been on dimethyl fumarate and discontinued 1-5 years prior to sample collection, 2 had previously been on teriflunomide and discontinued 1-3 years prior to sample collection, and 2 had previously been on glatiramer acetate and discontinued 4-6 years prior to sample collection.

**Table 1:** Patient Clinical and Demographic Characteristics.

	RRMS (n = 33)	Control (n = 22)
**Age (years; mean ± SD)**	47.4 ± 9.9	47.2 ± 11.7
**Sex [#(%)]**		
Female	26 (78.8)	17 (77.3)
Male	7 (21.2)	8 (22.7)
**EDSS**		
Range	0-4	
Median (IQR)	1.5 (2)	
**Disease Duration (years; mean ± SD)**	14.5 ± 9.49	

Venous blood was drawn into BD Vacutainer^®^ K2 EDTA tubes and plasma was obtained following 300 x g centrifugation for 10 minutes. Plasma was aliquoted, immediately frozen at -80°C for ~24 hours, and then transferred to liquid nitrogen for long-term storage. All samples were processed within 6 hours of collection. Blood was also drawn to obtain PBMCs using Ficoll density gradient centrifugation (ThermoFisher) and SepMate™ Tubes (StemCell Technologies) as per manufacturer’s instructions. Once isolated, PBMCs were cryopreserved and stored in liquid nitrogen. Prior to experimentation, cells were thawed quickly and immediately fixed and stained for flow cytometry.

### Nanotracking Analysis

To obtain particle concentration within plasma samples, nanotracking analysis (NTA) was performed. Plasma samples were diluted 1:1000 in 0.1µm filtered PBS and analyzed by NanoSight NS3000 (Malvern Panalytical, Malvern UK) equipped with a sCMOS camera and 488nm laser using NanoSight NTA software version 3.4. Hardware and analysis settings were as follows: Laser type: Blue488, Camera Level: 13, Camera gain: 3, Slider Shutter: 1232, Slider Gain: 219, Frame Rate: 25.0 FPS, Temperature: 25.0°C, Detect Threshold: 4, Blur size: auto, Max Jump Distance: auto.

### Plasma Staining Protocol

Cryopreserved plasma samples were thawed to room temperature on the benchtop and centrifuged for 20 minutes at 2,000 x g prior to experimentation. Based on particle concentration measurements obtained by NTA, the volume of sample stained represented ~1x10^9^ particles in a final volume of 5µL with 0.1µM filtered PBS. Prior to staining, all antibodies were centrifuged at 12,500 x g for 10 minutes to remove any antibody aggregates. Optimal antibody concentration was determined for each antibody based on previously published protocols and internal optimization studies (**Supplementary Figure 1**). Concentrations used ranged from 12.5-50ng antibody per ~10^9^ particles (**Supplementary Table 1**). Samples were stained for 1 hour at room temperature. To establish optimal gating parameters, negative staining controls consisting of equal volumes of 0.1µm filtered PBS and plasma samples were also stained (**Supplementary Figure 2**), and staining specificity was confirmed by treating stained samples with 1x Triton-X 100 to lyse EVs (19) (**Supplementary Figure 3**). Unless otherwise specified, prior to data collection, all samples were diluted to a final concentration of 1x10^8^ particles/mL in 0.1µm filtered PBS.

### EV Flow Cytometry

All experiments were designed following relevant criteria based on instrument choice and study/analysis design according to the MIFlowCyt-EV framework (**Supplementary Table 2**) (19).

EVs in stained plasma samples were quantified by flow cytometry using a CytoFLEX (Beckman Coulter) configured to Violet (405nm) side scatter (SCC) as the trigger parameter, with the threshold set at 800, as described previously (6). These settings were tested using a cocktail of size-calibrated beads (ApogeeMix for Flow Cytometer Performance Assessment (Apogee)) and recorded for 30 seconds. The V-SSC detector gain was set at 100 and phycoerythin (PE) fluorescence was measured using a 585/42nm bandpass filter with a detector gain set at 340; settings were chosen based on general guidelines for threshold and gain settings (5) and on balancing between minimizing noise and using 0.1µm filtered PBS as a background control. PE was chosen as the fluorophore for all markers based on its size, brightness, and ability to separate cleanly from background (20). Samples were run for 10 minutes at the slow flow rate (10µL/min). PE positivity was determined by setting gates based on the unstained plasma sample, and the plasma samples stained with anti-CD9-PE and anti-CD45-PE (**Supplementary Figure 4**). The EV gate was set independently for each plasma sample and remained constant for the analysis of all cell markers.

### PBMC Staining and Flow Cytometry

PBMC flow cytometry was performed on a majority subset of the cohort based on sample availability. PBMCs (200,000 cells) were stained with 1µL LIVE/DEAD™ fixable violet stain (Invitrogen/Thermo) in 1mL PBS and incubated for 30 minutes at 4°C in the dark. The cells were washed with 4mL PBS containing of 1% bovine serum albumin, 2mM EDTA, and 2mM sodium azide. Cells were decanted and resuspended in 100µL and added to a DURAclone IM Phenotyping BASIC tube (Beckman Coulter), mixed and incubated at 4°C for 30 minutes. The cells were washed and fixed with 100µL 2% paraformaldehyde. Data was acquired using the CytoFLEX (Beckman Coulter).

### Data Analysis

Flow cytometry data were analyzed using FlowJo™ v10.0 software (FlowJo, LLC, Ashland, OR). All statistical analyses were performed using Prism 9 (GraphPad Sofware Inc. San Diego, CA). No differences in overall CD45^+^ counts were observed between RRMS and healthy controls when normalized using CD9, therefore counts for lymphocyte subpopulations in both PBMC and EV analyses were normalized to CD45^+^ counts within each individual sample. For EV analyses, CD45^+^ and CD61^+^ counts were normalized to CD9^+^ counts. Outliers were identified using the ROUT method (21). Participants were deemed outliers and removed from all analyses if they were identified as such in measurements from two or more cellular markers; one individual was excluded from each group. Final cohorts consisted of 32 RRMS and 21 healthy controls. Data were analyzed using a Mann-Whitney test. Correlations between circulating EVs, PBMC populations, and clinical data were assessed using the Spearman correlation test. For all analyses, data are presented as mean ± SEM, unless otherwise noted, and α set to 0.05.

## Results

### Quantification of Plasma-Derived Extracellular Vesicles by Flow Cytometry

**Figure 1:**
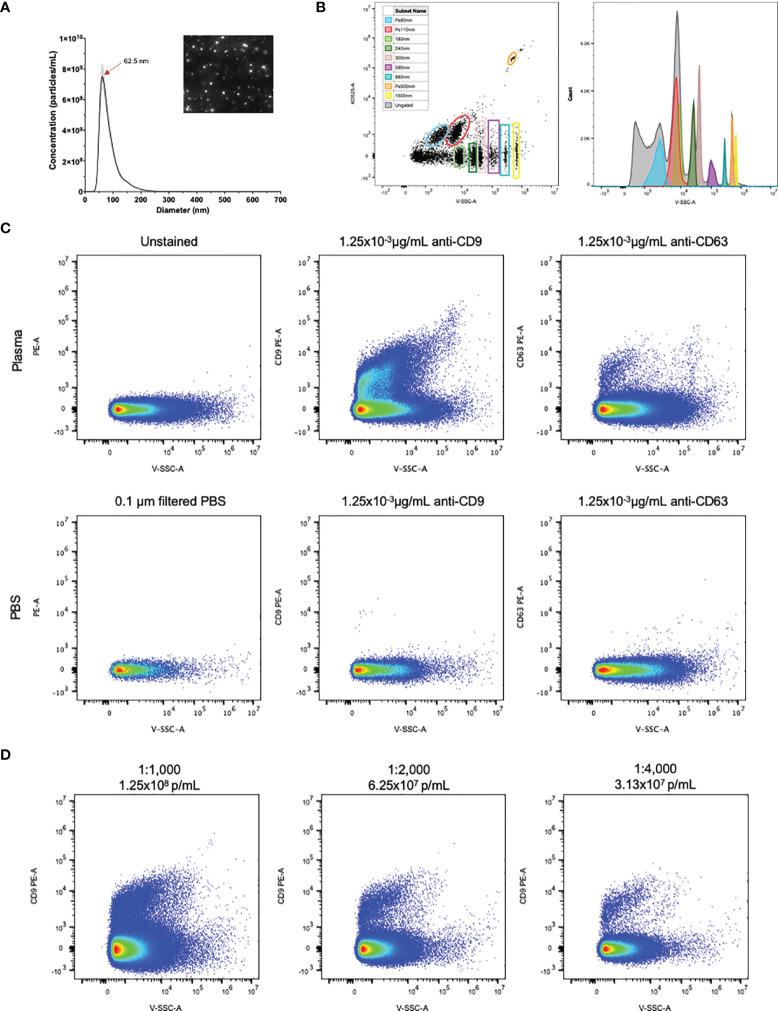
EVs in plasma are detectable by flow cytometry. **(A)** NTA particle size distribution with representative video screenshot (inset) of control human plasma. **(B)** Detection of size calibrated beads (Apogee) using violet side scatter (V-SSC-H) as the threshold parameter. Three of the bead populations we fluorescently labelled with the KO525 fluorophore and separated based on size (along the X axis) and fluorescence (along the Y axis). Histogram (right) reveals separation of all size beads based on V-SSC. **(C)** Positive staining of EVs labelled with antibodies against the EV markers CD9 and CD63, using antibodies diluted in 0.1µm filtered PBS as negative controls. **(D)** Plasma dilution series showing separation of PE positivity from background. Final antibody concentration was 0.019, 9.38x10^-3^ and 4.69x10^-3^ µg/mL for 1:1000, 1:2000 and 1:4000 dilutions.

NTA of healthy control human plasma revealed a size distribution with a single peak of 62.5nm (**Figure 1A**). While using flow cytometry calibrated for EV detection as previously described, individual populations of size calibrated beads ranging from 80-1,500nm are clearly visible (**Figure 1B**). Plasma samples were then stained with the EV markers CD9 and CD63 (22). Detection of CD9^+^ and CD63^+^ EVs are shown in **Figure 1C**, using both unstained plasma and antibody diluted in 0.1µm filtered PBS as background controls. Positive staining of CD9 on EVs in plasma was stronger and more consistent than CD63 positivity, therefore CD9 was chosen as the general EV marker for the duration of the study. To determine optimal particle concentration for the purpose of separating background from positive staining, a dilution series was conducted from 1.25x10^8^-3.13x10^7^p/mL (**Figure 1D**). Based on the results of the dilution series, 1.0x10^8^ was used as the final dilution factor for data collection.

### Increased Leukocyte Derived EVs Are Observed in RRMS Patient Plasma Compared to Healthy Controls

**Figure 2:**
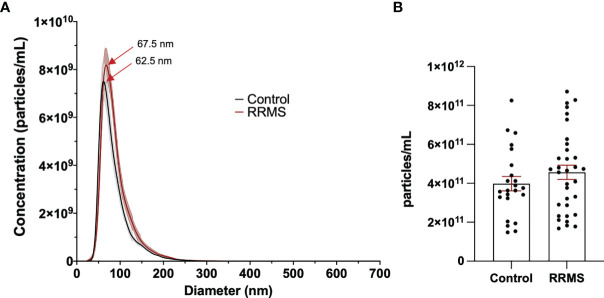
Comparison of EVs in control and RRMS plasma. **(A)** NTA size distribution of control and RRMS plasma. **(B)** Comparison of EV concentration measured by NTA between control and RRMS plasma samples.

Plasma from healthy control and RRMS patients was analyzed by NTA and displayed similar size distribution curves (**Figure 2A**). Plasma particle concentration measured in healthy control samples was 3.98x10^11^ ± 3.66x10^10^ p/mL and did not differ from RRMS samples, which were measured at 4.56x10^11^ ± 3.65x10^10^ p/mL (**Figure 2B**; p=0.28). Plasma particle concentration was not associated with age (Pearson’s r=0.226, p=0.326) or sex (p=0.107; data not shown)

**Figure 3:**
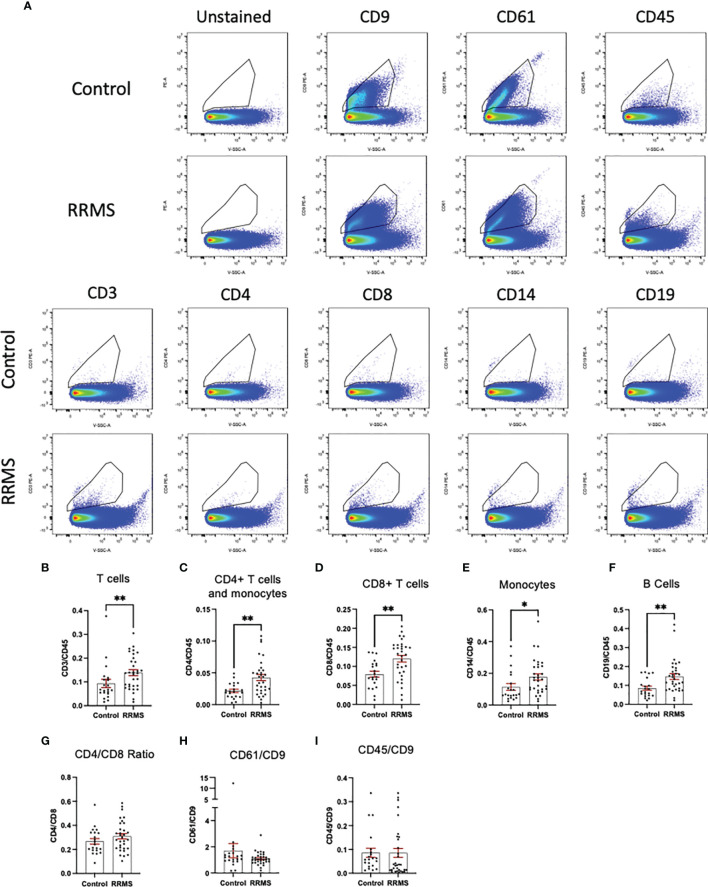
Comparison between RRMS and Control plasma reveals increased lymphocyte-derived EVs. **(A)** Representative flow cytometry plots displaying PE+ staining in plasma from control and RRMS cases. **(B-I)** Analysis of lymphocyte subsets normalized to CD45+ counts reveals significantly higher amounts of CD3+, CD4+, CD8+, CD14+ and CD19+ populations in RRMS compared to healthy controls. *p < 0.05 and **p < 0.01.

When comparing sub-populations of immune cell-derived EVs between healthy control and RRMS samples, we observed differences in all lymphocyte-derived populations investigated. **Figures 3A** displays representative flow plots of all markers analyzed in healthy control and RRMS samples. Significant increases were observed in RRMS compared to healthy control in the following populations (RRMS vs Healthy Control, **Figure 3**): CD3^+^ (0.139 ± 0.013 vs 0.093 ± 0.017; p=0.036), CD4^+^ (0.042 ± 0.005 vs 0.022 ± 0.003; p=0.002), CD8^+^ (0.120 ± 0.009 vs 0.079 ± 0.008; p=0.002), CD14^+^ (0.177 ± 0.019 vs 0.114 ± 0.021; p=0.013), and CD19^+^ (0.147 ± 0.015 vs 0.085 ± 0.011; p=0.002). No difference was observed between the CD4/CD8 ratio between RRMS and healthy control patients (**Figure 3G**; 0.309 ± 0.022 vs 0.267 ± 0.023, p=0.264). We also investigated ratios of platelet-derived EVs (CD61^+^) and all leukocyte-derived EVs (CD45^+^) normalized to CD9^+^ counts; no significant differences between RRMS and healthy control samples were observed for either CD61^+^ (**Figure 3H**, 1.099 ± 0.085 vs 1.705 ± 0.542, p=0.301) or CD45^+^ (**Figure 3I**; 0.085 ± 0.018 vs 0.086 ± 0.019, p=0.230).

### Levels of Lymphocyte Derived EVs in Circulation Do Not Correlate With Clinical and Demographic Variables in MS

Levels of circulating EVs displaying CD3, CD4, CD8, CD14 or CD19 were analyzed to identify correlations with age, EDSS, and disease duration in MS cases (**Table 2**). The only significant correlations observed were between CD14^+^ EVs and age (Spearman r=-0.391; p=0.03), and CD14^+^ EVs and disease duration (Spearman r=-0.362; p=0.045). However, age and disease duration were significantly correlated with one another (data not shown; Pearson’s r= 0.694; p<0.001). No correlations were observed between EV populations and age in the healthy control group (data not shown).

**Table 2:** Spearman correlation coefficients of lymphocyte EV populations with clinical and demographic variables in MS patients.

		Age	EDSS	Disease Duration
**CD3/CD45**	**r**	-0.182	-0.103	-0.105
	**p-value**	0.325	0.588	0.575
**CD4/CD45**	**r**	-0.107	-0.112	0.039
	**p-value**	0.568	0.548	0.834
**CD8/CD45**	**r**	-0.107	-0.134	-0.015
	**p-value**	0.566	0.48	0.938
**CD14/CD45**	**r**	-0.391	-0.186	-0.362
	**p-value**	0.03*	0.324	0.045*
**CD19/CD45**	**r**	-0.265	-0.165	-0.34
	**p-value**	0.15	0.383	0.061

*Indicates significance at α≤0.05.

### Immunophenotype of Circulating Leukocytes Are Not Correlated With Circulating EV Subpopulations

**Figure 4:**
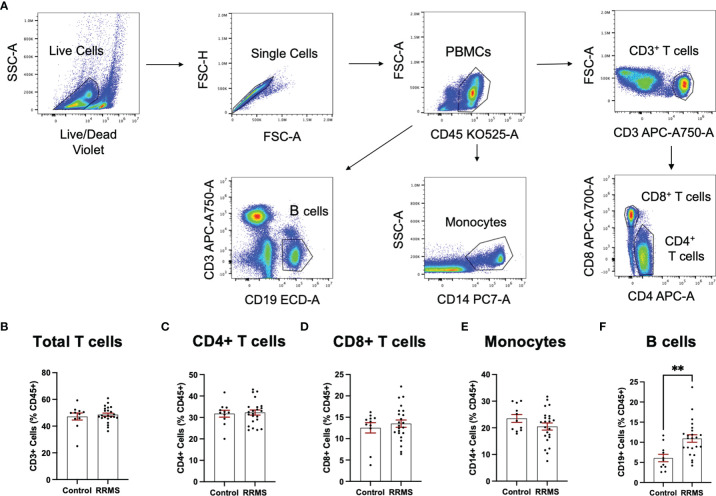
Circulating PBMC immunophenotypes in RRMS and controls. **(A)** Representative flow cytometry plots display the PBMC gating strategy used to quantify levels of circulating lymphocyte populations. No significant differences in circulating **(B)** total T cells, **(C)** CD4+ T cells and monocytes, **(D)** CD8+ T cells or **(E)** monocytes were observed between RRMS and controls. **(F)** a significantly increased number of CD19^+^ cells were observed in RRMS patients compared to controls **p < 0.01.

Circulating leukocytes from cryopreserved whole PBMC populations were quantified from a majority subset of the individuals included in the EV analysis (11 controls and 23 RRMS, selected based on sample availability). Populations of CD45^+^, CD3^+^, CD4^+^, CD8^+^, CD14^+^, and CD19^+^ cells were quantified by flow cytometry and displayed as %CD45^+^. Representative flow plots and gating strategies for PBMC quantification are displayed in **Figure 4A**. In whole PBMCs, no significant differences were observed between RRMS and healthy control cases for CD3^+^, CD4^+^, CD8^+^, or CD14^+^ cells (**Figures 4B–F**). A significant increase in CD19^+^ cells (**Figure 4F**; p=0.001) was observed in RRMS patients compared to healthy controls.

**Figure 5:**
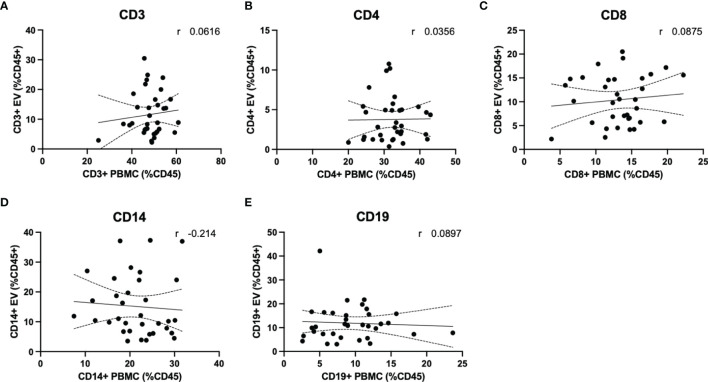
Circulating PBMC immunophenotype does not correlate with circulating lymphocyte-derived EVs. Plasma levels of EVs derived from **(A)** total T cells, **(B)** CD4+ T cells and monocytes, **(C)** CD8+ T cells, **(D)** monocytes and **(E)** B cells are not correlated with circulating levels of circulating parent lymphocyte populations.

Quantification of circulating lymphocytes were plotted against EVs displaying the same cellular markers, to determine whether increased levels of circulating EVs from specific lymphocyte populations were correlated with circulating levels of the parent cells. No significant correlations were observed between cells and EVs displaying any markers investigated in this study (**Figures 5A–E**). We also investigated whether a correlation existed when only RRMS cases were considered; no significant correlations were observed (data not shown).

## Discussion

The objective of this study was to evaluate circulating EVs secreted from MS-relevant lymphocyte populations, including T cells, B cells, and monocytes, and to determine whether these populations differed from healthy controls. Herein, we document that despite only limited changes to levels of circulating lymphocytes between RRMS and healthy control cases, significant increases in EV populations secreted from major lymphocyte populations were measured in untreated RRMS patients with low disability.

Flow cytometry has long been used as a method of quantifying and phenotyping cell populations. In addition to cells, larger EVs have also been quantified using this technique in many body fluids, including plasma and CSF (14, 16, 17, 23, 24). However, the full size range of EVs (30-1,000nm) extends within the level of background noise of conventional cytometers, creating a challenge of resolving a true range of positive signals (3, 25). The CytoFLEX instrument was specifically designed to mitigate this challenge by incorporating innovative optics, which results in low background and high signal (6). In addition, the 405nm laser for light scatter detection results in higher scatter from smaller particles further increasing signal (6). Using this instrument, we were able to resolve polystyrene beads down to 80nm in diameter (**Figure 1B**). We also successfully resolved EVs labelled with the well-characterized EV markers CD9 and CD63 (**Figure 1C**), and cell-specific markers of several major leukocyte populations (**Figure 3A**).

When analyzing EV populations in patient and healthy control cases, we observed no overall differences between total particle size and concentration as measured by NTA (**Figure 2**). Despite this, we did observe a significant increase in EVs displaying immune cell-specific markers of T cells (both cytotoxic and helper), monocytes, and B cells (**Figures 3C–F**). No differences in the CD4^+^/CD8^+^ ratios between RRMS and healthy control cases (**Figure 3G**), nor in the amount of leukocyte-derived (CD45^+^) or platelet-derived (CD61^+^) EVs when normalized to the general EV marker CD9 (**Figures 3H, I**).

Three previous studies have measured levels of EVs derived from various leukocyte populations within the plasma of RRMS patients, each using a different instrument. An early investigation demonstrated that levels of plasma microparticles (MP) displaying CD45 or CD14 were not different between untreated RRMS and healthy controls (14). Similar results were shown in two additional studies, which also observed no change in CD14^+^ MPs (16, 17). One of these studies reported no change in T-cell derived particles (CD3^+^) (16), which is supported by a more recent study that investigated subpopulations of T cells whereby no differences in CD4^+^ or CD8^+^ particles were measured between RRMS and control cases (17).

In contrast to these studies, we observed significant increases in EVs from all leukocyte populations investigated, namely T cells, B cells, and monocytes. Similar to our patient cohort, the aforementioned studies consisted of modest group sizes with low disability. Our RRMS cohort was larger by 9-17 individuals, depending on the study considered, which may have played a role in the discrepancies observed. Failure to detect these changes in the previous studies may be due, in part, to the instrumentation. For example, the most recent study, performed by Groen and colleagues, specify their instrument was capable of differentiating particles >200nm in diameter (17), which is likely to omit data from smaller EVs. In the current study, the instrument used to collect data uses novel technologies to optimize small particle detection (6). Our NTA and flow cytometry data, which is consistent with others using the CytoFLEX, demonstrates that smaller diameter EVs (<100nm) are present in bodily fluids and can be readily phenotyped. The CytoFLEX can resolve beads of known diameters <100nm suggesting that we are also able to detect EVs smaller than 100nm (**Figure 1B**) (6). Based on theoretical calculations, Brittain and colleagues surmise the CytoFLEX can detect EVs down to 12nm in diameter, and that EVs in the 30nm diameter range cluster around the 60nm polystyrene bead (6). Since the earlier studies were unable to resolve the smaller vesicle populations, it could be this population that is driving the differences we observe in particles derived from leukocyte populations.

An important finding of the current study is that despite observing no changes in levels of circulating T cells or monocytes between healthy control and RRMS cases (**Figure 4**), we did see a significant increase in circulating EVs bearing cell-specific markers for all these cell populations (**Figures 3B–F**). Our cellular phenotyping data is consistent with a previous report from a study with similar cohort size, which documented no major changes in the levels of circulating leukocyte populations in MS compared to healthy controls (26). The only exception being that we found a significant increase in circulating CD19^+^ B cells in RRMS (**Figure 4F**). Recent evidence suggests that circulating levels of CD19^+^ B cells in MS cases is heterogenous (27). Therefore, the increase in B cells observed in the current study may be due to unintentional random sampling of a population of MS cases skewed towards higher levels of B cells. Additional investigations into B cell populations in MS with larger cohorts are indeed needed before concluding whether differences in circulating B cells truly exist between RRMS and healthy control cases. In this study, we also noted a significant increase in CD19^+^ B cell-derived EVs, however, despite also observing increased CD19^+^ B cells, these levels were not correlated (**Figure 5E**), suggesting that the increase in CD19^+^ EVs is not likely a product of increased levels of CD19^+^ cells within circulation, but perhaps rather due to activation status.

Our results suggest that despite not observing differences in the levels of circulating immune cell subsets, EVs derived from these cells are elevated in RRMS compared to healthy controls. Previous studies have provided evidence suggesting that cells release more EVs under pro-inflammatory vs. control conditions and can transfer inflammatory signals *via* EVs to neighboring cells, and even cells distant in the body (24, 28). While our results do not directly show that this propagation occurs, they allude to a possible mechanism whereby a cells’ pro-inflammatory activities can propagate in RRMS cases *via* secretion of EVs. Further investigations into the functional relevance of these EV populations within the circulation in RRMS will provide interesting insights into whether specific EVs can drive pathological activity in these otherwise non-inflamed cases with low disability.

Diagnosing MS is complex and involves multiple invasive and expensive procedures often including magnetic resonance imaging (MRI), and a lumbar puncture (8). Additionally, with the diagnostic criteria requiring dissemination in both space and time, patients are often left undiagnosed for extended periods of time (18). The drive to identify more specific biomarkers for MS is active but remains unmet. The identification of blood-based biomarkers is of particular interest, as sample collection is simple, cost effective and non-invasive. Complex patterns of circulating EVs and their contents could prove valuable for identifying markers of complex conditions like MS (3). Secreted EVs can cross the blood-brain-barrier (BBB) and can transfer inflammatory signals to recipient cells in the absence of additional inflammatory triggers or BBB breakdown (24, 28). Therefore, immune-derived EVs from blood and/or CSF can also possibly inform on potential ongoing pathological processes in the CNS or the periphery in the absence of obvious changes to a patient disability or concrete demyelinating event. In fact, recent evidence suggests that levels of myeloid cell-derived EVs in the CSF may serve as predictive biomarkers for disease course and disability accrual in MS (23). Whether this extends to myeloid cell-derived EVs measured in the periphery has yet to be investigated and may represent an important metric in blood to consider.

Further investigation into this hypothesis using larger group sizes and cases with increased level of disability is, of course, required before the utility of leukocyte derived EVs as a biomarker for MS is fully understood. It will be important to determine whether these changes persist or change with relapse activity or progression, and whether they are sensitive to specific DMT use. Our data suggest that levels of plasma EVs do not correlate with disability as measured by EDSS (**Table 2**), but the limited disability of our sample may have prevented identification of any true associations that may exist. This study was limited to using EDSS as a measure of disability, as we did not have access to sufficient MRI data. It will be critically important for future studies to determine whether EV populations are associated with other more objective markers of disability and disease activity, including MRI and neurofilament light chain measures.

This study provides evidence that plasma levels of EV populations secreted from T cells, B cells, and monocytes are elevated in untreated RRMS cases with low disability, despite no change to inflammatory activity in these patients as measured by PBMC flow cytometry. These results suggest a possible future clinical utility of measuring circulating EV populations as a biomarker in MS. While the current study provides an important initial step in this direction, future studies investigating circulating EVs during the time of MS diagnoses, and longitudinally throughout the disease process are needed.

## Data Availability Statement

The original contributions presented in the study are included in the article/**Supplementary Material**. Further inquiries can be directed to the corresponding author.

## Ethics Statement

The studies involving human participants were reviewed and approved by Health Research Ethics Authority. The patients/participants provided their written informed consent to participate in this study.

## Author Contributions

SB participated in data collection, analysis, interpretation and prepared the original manuscript. NF participated in data collection. CC, SB participated in instrumentation assistance and assay development/optimization. CM participated in developing original project concept, data collection, analysis, and interpretation, and preparing and submitting the original manuscript. All authors contributed to the article and approved the submitted version.

## Funding

The author(s) disclose receipt of the following financial support for the research, authorship and/or publication of this article; this study was funded by the Multiple Sclerosis Society of Canada (CM: EGID#3499) and the Canadian Institute for Health Research (CM: PJT155933). SB is gratefully supported by a doctoral studentship award from the Multiple Sclerosis Society of Canada.

## Conflict of Interest

The authors declare that the research was conducted in the absence of any commercial or financial relationships that could be construed as a potential conflict of interest.

## Publisher’s Note

All claims expressed in this article are solely those of the authors and do not necessarily represent those of their affiliated organizations, or those of the publisher, the editors and the reviewers. Any product that may be evaluated in this article, or claim that may be made by its manufacturer, is not guaranteed or endorsed by the publisher.
