# Supplementary material for: Analysis of Plasma Using Flow Cytometry Reveals Increased Immune Cell-Derived Extracellular Vesicles in Untreated Relapsing-Remitting Multiple Sclerosis
Source: Front Immunol. 2022 Mar 22;13:803921. doi: 10.3389/fimmu.2022.803921 (PMC8980610; doi:10.3389/fimmu.2022.803921)
Supplement: Supplementary file 1 [file DataSheet_1.docx]

**Supplementary Table 1:** Antibodies used in phenotypic analysis of EVs. All antibodies were purchased from BD Biosciences, except for CD63-PE which was purchased from Beckman Coulter.

**Supplementary Table 2**: MIFlowCyt-EV framework checklist.

| **Framework Criteria** | **Please complete each criterion** |
| --- | --- |
| 1.1 Preanalytical variables conforming to MISEV guidelines. | Relevant preanalytical variables are detailed in the methods section of the manuscript. |
| 1.2 Experimental design according to MIFlowCyt guidelines. | MIFlowCyt checklist items 1.1, 1.2, and 1.3 are available in the manuscript |
| 2.1 Sample staining details | Sample staining procedure is recorded in the methods. |
| 2.2 Sample washing details | Samples were not washed. Instead, unbound antibodies were diluted to a final concentration where they contributed minimally to positive fluorescence. See supplementary figure 1 for this process |
| 2.3 Sample dilution details | Final dilutions and diluents used are recorded in the methods |
| 3.1 Buffer alone controls. | Acquisition settings remained consistent throughout the duration of data collection. Buffer-only controls were ran and analysed once for each day of collection. All samples were recorded for 10 minutes. |
| 3.2 Buffer with reagent controls. | Buffer with reagent controls were not ran and analysed with every sample, however, they were ran sporadically throughout the data collection phase, and always recorded and analysed using the same settings and concentration as test samples. Buffer with reagent controls resulted in negligible positive staining (supplementary figure 2 and figure 1). |
| 3.3 Unstained controls. | unstained controls of each sample were recorded and analyzed at the same settings during the same experiment as stained samples. |
| 3.4 Isotype controls. | Isotype controls were not used or analyzed. |
| 3.5 Single-stained controls. | All experiments in this manuscript contained only single stains. |
| 3.6 Procedural controls. | Buffer only and buffer with reagent controls were treated procedurally in an identical manner as test samples. |
| 3.7 Serial dilutions. | Serial dilutions were conducted early on while determining optimal final particle concentration for analysis. Once 1x10^8 particles/mL was chosen as a final concentration serial dilutions were not performed as the concentration was standardized across individuals. |
| 3.8. Detergent treated EV-samples | Triton-X was used as a detergent control; See supplementary figure 3. |
| 4.1 Trigger Channel(s) and Threshold(s). | Relevant details are described in Materials and methods. |
| 4.2 Flow Rate / Volumetric quantification. | Flow rate was set to 10µL/min (slow flow rate) using the CytEXPERT software. Calibration was not performed |
| 4.3 Fluorescence Calibration. | Fluorescence calibration as not performed. |
| 4.4 Light Scatter Calibration. | Light scatter calibration was not performed |
| 5.1 EV diameter/surface area/volume approximation. | EV diameter, surface area or volume was not estimated using flow cytometry. |
| 5.2 EV refractive index approximation. | EV refractive index was not approximated |
| 5.3 EV epitope number approximation. | EV epitope was not approximated |
| 6.1 Completion of MIFlowCyt checklist. | Details are available in the manuscript. |
| 6.2 Calibrated channel detection range | Fluorescence or light scatter calibration was not performed |
| 6.3 EV number/concentration. | EV number/concentration was not reported as a raw number, but instead normalized to the CD45+ count obtained from the same patient sample. Total particle concentration in each sample was obtained by nanotracking analysis. |
| 6.4 EV brightness. | EV brightness was not evaluated |
| 7.1. Sharing of data to a public repository. | The original contributions presented in the study are included in the article/supplementary material, further inquiries can be directed to the corresponding author/s. |
|  |  |
|  |  |
|  |  |
|  |  |
|  |  |

**Supplementary Figure 1: Representative antibody titration series to determine optimal concentration.** A) Background instrument noise was assessed with 0.1um filtered PBS and unstained plasma at a final concentration of 1x10^8^ particles/mL in 0.1um filtered PBS B) Representative antibody titration series for anti-CD9 PE. Each antibody was tested at a final concentration range of 4.16x10-3 to 5.2x10-4 ug/mL to assess background when diluted in PBS alone, and signal when staining plasma samples (1x10^8^ particles/mL). C) Gated PE+ counts show a linear relationship between PE+ events in both stained and unstained samples. For this antibody, a final concentration of 1.25x10^-3^ ug/mL was chosen based on minimum noise generation and sufficient positive staining. This process was repeated for all antibodies used in this study. Plots displayed here represent 2-minute captures at a flow rate of 10uL/min.

**Supplementary Figure 2: Antibody in 0.1um filtered PBS Negative Controls.** Based on results of antibody optimization final concentrations of each antibody were diluted in 0.1um filtered PBS to confirm minimal contribution of unbound antibodies and antibody aggregates to background PE fluorescence. Plots displayed in this figure represent 10-minute captures at a flow rate of 10uL/min.

**Supplementary Figure 3: Triton-X Negative Control.** Representative series showing CD9-PE+ events (middle) are eliminated with treatment of plasma with 1x Triton-X (right).

**Supplementary Figure 4: Gating Strategy**. A) Stepwise addition of the gate beginning by setting the lower limit based on the unstained sample, followed by the left angled limit based on CD45+ antibody aggregates and the top/left limits based on CD9 stained sample. B) Application of the gate to a representative sample illustrating the total number of positive events counted. Plots here represent 10-minute captures at a flow rate of 10uL/min
